# A Strategy for Rapid Acquisition of the β-D-Fructofuranosidase Gene through Chemical Synthesis and New Function of Its Encoded Enzyme to Improve Gel Properties during Yogurt Processing

**DOI:** 10.3390/foods12081704

**Published:** 2023-04-19

**Authors:** Zhou Chen, Yimei Shen, Jiangqi Xu

**Affiliations:** Beijing Technology and Business University, Beijing 100048, China

**Keywords:** gel properties, β-D-fructofuranosidase, *Aspergillus luchuensis*, fructooligosaccharides, prebiotics

## Abstract

A chemical gene synthesis strategy was developed in order to obtain β-D-fructofuranosidase, and a novel gene, AlFFase3, was characterized from *Aspergillus luchuensis* and expressed in *Escherichia coli*. The recombinant protein was purified, showing a molecular mass of 68.0 kDa on SDS-PAGE, and displaying a specific activity towards sucrose of up to 771.2 U mg^−1^, indicating its exceptional enzymatic capacity. AlFFase3 exhibited stability between pH 5.5 and 7.5, with maximal activity at pH 6.5 and 40 °C. Impressively, AlFFase3, as a soluble protein, was resistant to digestion by various common proteases, including Flavourzyme, acidic protease, pepsin, neutral protease, Proteinase K, alkaline proteinase, and trypsin. AlFFase3 also demonstrated significant transfructosylation activity, with a yield of various fructooligosaccharides up to 67%, higher than almost all other reports. Furthermore, we demonstrated that the addition of AlFFase3 enhanced the growth of probiotics in yogurt, thereby increasing its nutritional value. AlFFase3 also improved the formation of yogurt gel, reducing the gel formation time and lowering the elasticity while increasing its viscosity, thereby improving the palatability of yogurt and reducing production costs.

## 1. Introduction

Functional foods are products that are specifically formulated to improve health, prevent/treat diseases, and promote overall well-being [1]. Fructooligosaccharides (FOSs) are a bioactive ingredient widely used in functional foods. FOSs are composed of linear fructose units linked by β (2-1) bonds, ranging from 2 to 60 units, and are attached to a terminal glucose residue. FOSs have gained increased attention in recent years due to their numerous health benefits. Chronic consumption of FOSs has been reported to reduce serum triglyceride and cholesterol levels, improve the absorption of ions such as Ca^2+^ and Mg^2+^, inhibit the growth of pathogenic microorganisms in the intestine, and act as prebiotics that have positive effects on beneficial bacteria strains, such as *Bifidobacterium* and *Lactobacillus*, in the colon [2]. In the food industry, FOSs, as well-established prebiotics, can be used to supplement dairy products, such as yogurt, resulting in improvements in the microbiological and physicochemical properties of these products [3].

Various approaches have been developed to enhance fructooligosaccharide (FOS) production, and enzymatic catalysis has emerged as an effective means for achieving this goal. Currently, fructosyltransferases (EC 2.4.1.9) and β-fructofuranosidases (FFases) exhibiting high transfructosylation activity (EC 3.2.1.26) are widely utilized for FOS production [4]. Synthesis via β-fructofuranosidase is considered comparatively straightforward, as it does not require activated substrates, and benefits from the widespread availability of β-fructofuranosidase. Microbial β-fructofuranosidases, such as those sourced from *Aspergillus* spp., *Bifidobacterium* spp., *Bacillus* spp., and *Candida* spp. are preferred for commercialization. Although various *Aspergillus* spp. have been reported to produce β-fructofuranosidases, such as *A. brasiliensis* [5], *A. tamari* [6], *A. oryzae* [2], and *A. niger* [7], those exhibiting high transfructosylation activity have been rare, which limits their application in FOS production. Therefore, discovering novel *Aspergillus* FFases with high transfructosylation activity investigating their transfructosylation reactions are essential to achieve optimal yields of high-quality FOSs for industrial use [8]. Some *Aspergillus* spp., such as *A. thermomutatus* [9], *A. awamori* [4], and *A. oryzae* [10,11], produce FFases with transfructosylation activity, and their production, immobilization, and FOS synthesis have been studied. Based on these preliminary reports, *Aspergillus* β-fructofuranosidases with high transfructosylation activity exhibit great potential for FOS synthesis. However, identifying additional enzymes of this type, as well as β-fructofuranosidases possessing novel properties, is desirable.

This study presents the discovery of a novel β-fructofuranosidase gene found in *A. luchuensis*, as well as the successful expression, purification, and characterization of the enzyme. The transfructosylation activity of the enzyme was also evaluated, along with its potential for application in yogurt production, such as improving nutritional properties and gel formation.

## 2. Materials and Methods

### 2.1. Materials and Reagents

A glucose oxidase kit was procured from Applygen Technologies Inc. (Beijing, China). Ni-IDA columns were acquired from GE Life Sciences (Pittsburgh, PA, USA). Silica gel plates (60F 254) were obtained from Merck KGaA (Darmstadt, Germany).

### 2.2. Gene Synthesizing, Cloning, and Expression

An uncharacterized protein-coding gene (GenBank accession no: XP_041545449.1) from *A. luchuensis* was retrieved from GenBank and codon-optimized for expression in *E. coli*. The optimized gene fragment (named *AlFFase3*) was chemically synthesized by Synbio Technologies (China) and amplified by PCR using primers with *NdeI* and *XhoI* restriction sites (underlined): AlFFase3-up (ATTCTACATATGATGAAATTACAAACTGCTTCTGTT) and AlFFase3-down (ATTCCGCTCGAGTCAGTATTGCCGATCAGGCCAAGC). The purified PCR product was digested with *NdeI* and *XhoI*, and then ligated into vector pET-28a(+). All recombinant plasmids used were sequenced and verified.

Subsequently, pET-28a(+)-AlFFase3 was transformed into competent *E. coli* BL21 (DE3) cells for protein expression. The cells were cultured in Luria–Bertani medium supplemented with 50 µg/mL kanamycin at 37 °C until the optical density reached 0.6–0.8. Protein expression was then induced by adding 1 mM isopropyl β-D-thiogalactopyranoside, and the cells were cultured further at 20 °C for 12 h. The cells were then harvested through centrifugation at 10,000× *g* for 10 min at 4 °C.

### 2.3. Purification and Sodium Dodecyl Sulfate-Polyacrylamide Gel Electrophoresis (SDS-PAGE) Analysis of Recombinant AlFFase3

The harvested cells were resuspended in buffer A (50 mM Tris-HCl, pH 7.4) and sonicated to disrupt the cells. The resulting crude enzyme-containing supernatant was obtained through centrifugation at 10,000 rpm for 10 min. The target enzyme was purified using a 0.8 × 10 cm Ni-IDA column. The column was equilibrated with buffer B (buffer A containing 20 mM imidazole), and the crude cell extract was loaded onto the column. Unbound or weakly bound proteins were removed by washing the column with buffer C (buffer A containing 50 mM imidazole) at a flow rate of 1.0 mL/min. The target protein was then eluted with buffer D (buffer A containing 200 mM imidazole). The purity of the target protein was evaluated by 12.5% SDS-PAGE, stained with Coomassie Brilliant Blue R-250. Molecular weight standards, including rabbit phosphorylase B (97.4 kDa), bovine serum albumin (66.2 kDa), rabbit actin (43.0 kDa), bovine carbonic anhydrase (31.0 kDa), trypsin inhibitor (20.1 kDa), and hen egg white lysozyme (14.4 kDa), were utilized [12].

### 2.4. Enzyme Assay and Protein Determination

A total of 20 µL of enzyme solution (AlFFase3) was mixed with 180 µL of substrate (200 mg/mL sucrose) and incubated at 40 °C for 10 min. The β-D-fructofuranosidase activity was determined by measuring the amount of glucose released using a glucose oxidase kit. One unit of β-D-fructofuranosidase activity was defined as the enzyme amount that released 1 μmol of glucose per minute. The protein concentration was determined by the Lowry method, with bovine serum albumin as the standard [13].

### 2.5. Characterization of the Recombinant β-D-Fructofuranosidase AlFFase3

The optimal pH and temperature for β-D-fructofuranosidase activity of AlFFase3 were investigated using sucrose as the substrate. Buffers with varying pH values, ranging from 3.0 to 10.5, including sodium citrate (pH 3.0–6.0), MES (pH 5.0–6.5), sodium phosphate (pH 6.0–8.0), MOPS (pH 6.5–8.0), Tris-HCl (pH 7.5–9.0), and glycine-NaOH (pH 9.0–10.5), were utilized. Additionally, the activity of AlFFase3 was assayed at a temperature range of 25–70 °C.

The stability of AlFFase3 was assessed under varying acidic and basic conditions, as well as at different temperatures. The enzyme solution was incubated in the aforementioned buffers at 40 °C for 30 min, or at temperatures ranging from 30 to 55 °C for 30 min. Subsequently, all treated proteins were rapidly cooled to 0 °C, and their residual activities were determined using the standard enzyme assay.

Additionally, the capacity of AlFFase3 to withstand cations that could potentially exist in complex application environments was evaluated. The enzyme was incubated with 1 mM cation at 40 °C in 50 mM sodium phosphate buffer (pH 6.5) for 30 min. The residual activity of the treated protein was then measured using the standard assay.

The influence of different metal ions and reagents, including Mg^2+^, Fe^3+^, Mn^2+^, Fe^2+^, Co^2+^, Ca^2+^, Zn^2+^, Al^3+^, Cu^2+^, EDTA, and SDS, on AlFFase3 was also determined. AlFFase3 was incubated with 1 mM of various metal ions at 40 °C in sodium phosphate buffer (50 mM, pH 6.5) for 30 min, and then their residual activity was measured using the standard assay.

### 2.6. Resistance of AlFFase3 to the Protease Hydrolysis

In order to investigate the potential protease resistance of AlFFase3, various common proteases, including trypsin, pepsin, Proteinase K, acidic protease, neutral protease, alkaline proteinase, and Flavourzyme were selected for experimentation. AlFFase3 was mixed with each protease (10 U/mL) and incubated at 37 °C for 30 min. The residual β-D-fructofuranosidase activity of the treated enzyme, and of the control (without protease treatment), was determined using the standard assay.

### 2.7. New Function of Producing Various FOSs of AlFFase3

In order to assess the potential of AlFFase3 in the preparation of FOSs, a reaction mixture containing 10 U/mL of AlFFase3 and 20% (*w*/*v*) sucrose was incubated at 40 °C in 50 mM sodium phosphate buffer (pH 6.5). Aliquots were collected at regular intervals and heated in boiling water for 5 min. The samples were then placed onto silica gel plates and developed twice using a solvent system composed of n-butanol:acetic acid:water (2:1:1 *v*/*v*/*v*). Saccharides were visualized through heating the plates in an oven after spraying them with a mixture of methanol and sulfuric acid (95:5, *v*/*v*). The FOS yield was analyzed using high-performance liquid chromatography (HPLC) with a YMC-Pack ODS-AQ column (250 × 460 mm). The mobile phase consisted of ultrapure water and was run at a flow rate of 0.5 mL/min.

### 2.8. Effect on Gel Properties of AlFFase3 during the Processing of Yogurt

AlFFase3 was incorporated into the yogurt-making process in order to evaluate its impact on yogurt formation and gel properties. The mixture consisted of 0–10 U/mL of AlFFase3, 10% sucrose, 3% starter (Angel Yeast Co., Ltd., Yichang, China), and 30 mL of fresh milk, which was then fermented at 40 °C. A sample without AlFFase3 was used as a blank control. The gel properties of the yogurt, including the gel formation period, Elasticity Index, and Macroscosity Index, were subsequently evaluated using a rheometer.

## 3. Results and Discussion

### 3.1. Excavation, Cloning, and Expression of a β-D-Fructofuranosidase-Encoding Gene from A. luchuensis

**Figure 1:**
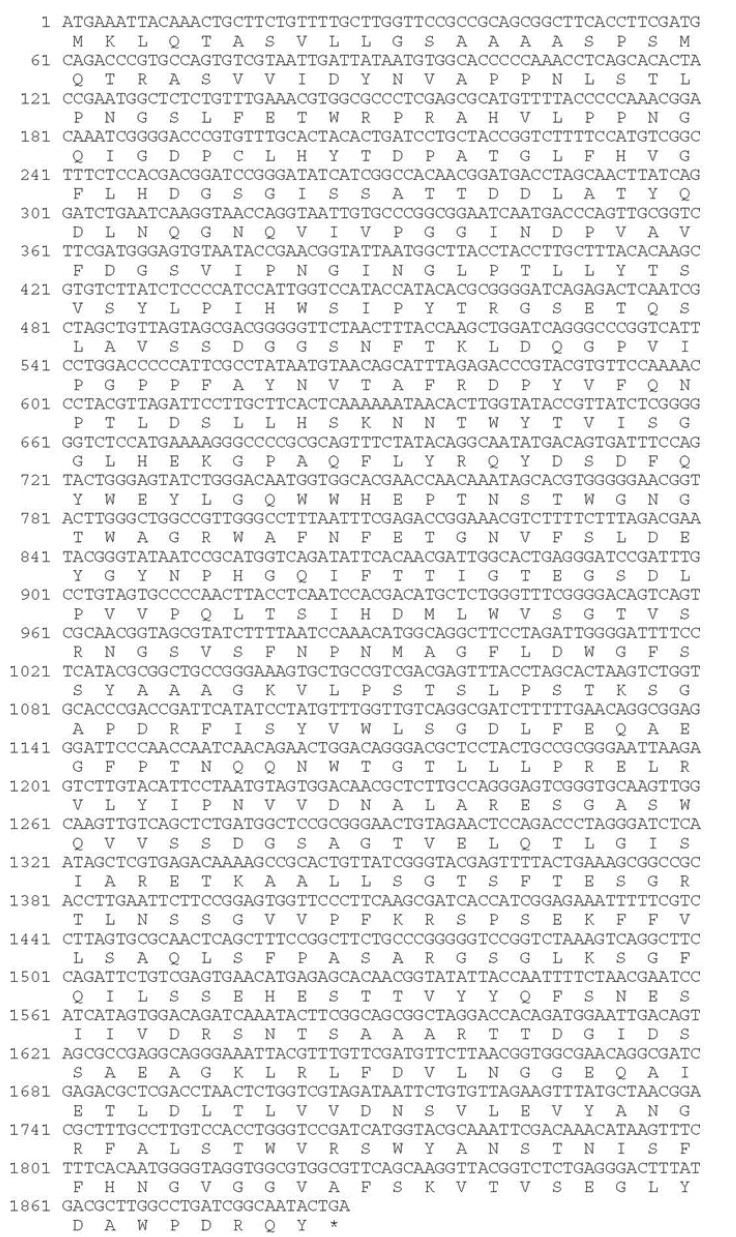
Nucleotide and deduced amino acid sequences of the full-length cDNAs and flanking regions of AlFFase3.

A newly registered gene, AlFFase3, which had not previously been characterized in the genome of *A. luchuensis*, was successfully evaluated. Figure 1 presents the amino acid and nucleotide sequences of AlFFase3, which has a full-length open reading frame of 1887 bp and encodes 628 amino acids. The predicted molecular mass of the protein is 67.9 kDa, with a deduced *p*I value of 4.70. The amino acid sequence of AlFFase3 was analyzed for its homology using the Basic Local Alignment Search Tool (BLAST). Results showed that the protein sequence of AlFFase3 exhibited the highest identity with β-D-fructofuranosidases/invertases from the same genus, such as *A. piperis* (GenBank no: XP_025509931.1), *A. tubingensis* (GenBank no: XP_035359123.1), and *A. neoniger* (GenBank no: XP_025484376.1), but lower identity with proteins from *Penicillium arizonense* (GenBank no: XP_022490399.1) and *Hypoxylon* sp. CI-4A (GenBank no: OTB01346.1).

Microorganisms are significant sources of commercial β-D-fructofuranosidases. Various microorganisms, including *Aspergillus* spp. [5,6,9], *Bifidobacterium* spp. [14], *Bacillus* spp. [15,16,17], *Candida guilliermondii* [18], *Microbacterium trichothecenolyticum* [19], and *Cunninghamella echinulata* [20], exhibit sufficient ability to produce β-D-fructofuranosidase. Among them, *Aspergillus* spp. is the best resource for β-D-fructofuranosidase production. β-D-Fructofuranosidases from *A. oryzae* [10], *A. awamori* [4], *A. sojae* [21], and *A. tamarii* [6] have been studied in terms of optimization, immobilization, purification, and biochemistry. However, most reports have focused on native enzymes, and these studies were limited by the specificity of the strains. In recent decades, with the rapid development of bioinformatics, more registered β-D-fructofuranosidases have become visible in specialized databases. Evaluating several high-value genes/amino acids from the databases and chemically synthesizing them directly has become a new and efficient strategy to obtain many target proteins. In this study, we screened a novel and uncharacterized protein-coding gene from *A. luchuensis*, successfully expressed it in *E. coli*, and produced recombinant β-D-fructofuranosidase for further investigation.

### 3.2. Purification, SDS-PAGE Analysis, and Enzyme Assay of the Recombinant β-D-Fructofuranosidase AlFFase3

**Figure 2:**
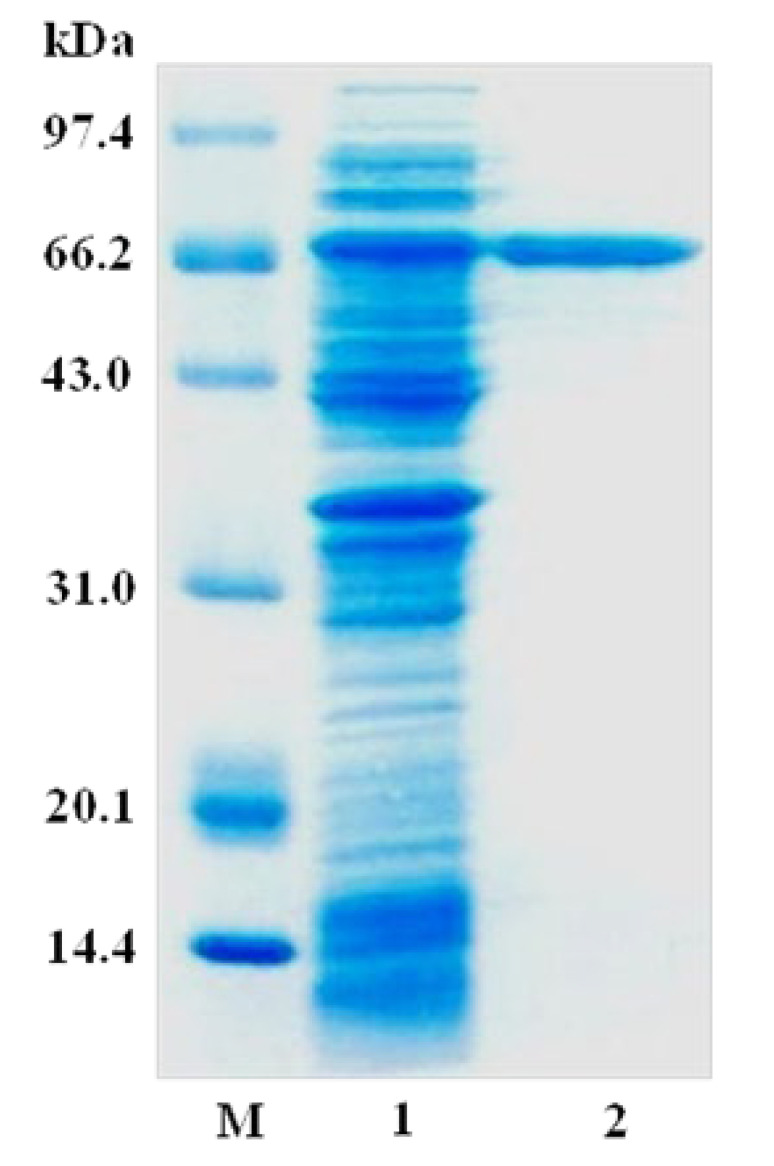
SDS-PAGE analysis of purified AlFFase3. Lane M—low molecular weight standard protein markers; lane 1—crude lysate; lane 2—purified AlFFase3.

The β-D-fructofuranosidase, AlFFase3, was successfully expressed and subsequently purified using Ni-IDA affinity chromatography. SDS-PAGE analysis revealed a single band at 68.0 kDa, in (Figure 2). Based on homology analysis, AlFFase3 was identified as a β-D-fructofuranosidase, which was further confirmed by the observed enzyme activity. The purification process is summarized in Table 1, and one-step purification resulted in a significant increase in specific activity of AlFFase3, from 138.2 U/mg to 771.2 U/mg.

According to the available literature, the sizes of *Aspergillus* β-D-fructofuranosidases exhibit variability. For instance, β-D-fructofuranosidases from *A. oryzae* S719 (95 kDa) [2], *A. niger* (116 kDa) [7], *A. sojae* JU12 (35 kDa) [15], and *A. terreus* (32 kDa) [6] all have different molecular masses from that of AlFFase3. However, the molecular mass of AlFFase3 is similar to that of most reported bacterial β-D-fructofuranosidases, such as several bifidobacterial β-fructofuranosidases, which range from 59 to 75 kDa.

β-D-fructofuranosidases from the *Aspergillus* species have been widely used as competitive invertases in various biotechnological applications, due to their exceptional specific activity. For instance, *A. terreus* and *A. sojae* exhibited particularly high specific activities of 1985.7 U/mg and 1886.3 U/mg, respectively, as reported by de Almeida et al. and Lincoln & More [15,22]. In this study, the specific activity of AlFFase3 was determined to be 771.2 U/mg, which is lower than the specific activities of these two *Aspergillus* species. However, compared to several reported bacterial β-D-fructofuranosidases, such as those from *Microbulbiferr* FF33 (685.6 U/mg) [23], *Leuconostoc mesenteroides* (469 U/mg) [24], and *Microbacteriumtri chothecenolyticum* (225 U/mg) [19], AlFFase3 exhibited greater hydrolytic activity towards sucrose, indicating its potential in industrial applications.

### 3.3. Characterization of Recombinant AlFFase3

**Figure 3:**
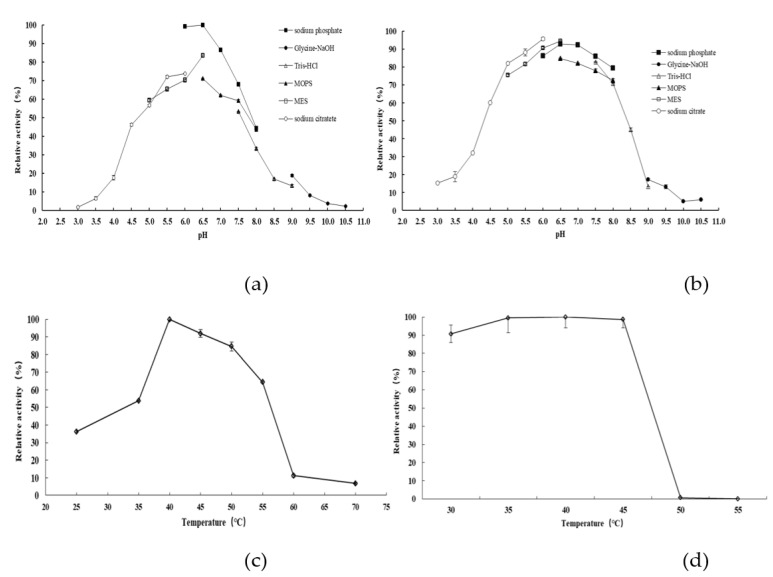
pH and temperature profiles of AlFFase3. Effect of pH on activity (**a**) and stability (**b**) was performed at 40 °C in 50 mM different buffers: sodium citrate (○), MES (□), sodium phosphate (■), MOPS (▲), Tris-HCl (△), and glycine-NaOH (●). The remaining activities were measured after incubation for 30 min at 40 °C, over various pH ranges. Effect of temperature on the activity (**c**) and thermostability (**d**) of AlFFase3 were determined at temperatures ranging from 25 °C to 70 °C in 50 mM sodium phosphate buffer (pH 6.5).

The recombinant β-D-fructofuranosidase AlFFase3 was subjected to characterization of its biochemical properties, which included determination of its optimal reaction pH and temperature, pH stability and thermostability, and the effects of various metal ions and reagents on its activity. AlFFase3 exhibited maximum β-D-fructofuranosidase activity at pH 6.5 (sodium phosphate buffer) (Figure 3a) and at a temperature of 40 °C (Figure 3c). However, acidic or basic environments and high temperature had an adverse effect on the stability of this recombinant β-D-fructofuranosidase. Nevertheless, after incubation for 30 min, AlFFase3 retained over 70% of its maximal catalytic activity at pH 5.0–8.0 (Figure 3b) and at temperatures up to 45 °C (Figure 3d). Certain cations, such as Fe^3+^ (162.5%), Mg^2+^ (110.3%), Fe^2+^ (109.2%), and SDS (112.3%), enhanced the enzymatic activity of AlFFase3, while other ions, such as Zn^2+^ (20.0%), Cu^2+^ (50.6%), Co^2+^ (60.7%), Ca^2+^ (85.0%), and ethylenediaminetetraacetic acid (EDTA; 86.0%), inhibited this β-D-fructofuranosidase. Al^3+^ (101.2%) and Mn^2+^ (96.4%) had little effect on the activity of AlFFase3.

Bacterial β-D-fructofuranosidases have their highest activity within the pH range of 6.0–8.0, whereas those produced by fungi have an optimal reaction pH of 5.0–6.5. The β-D-fructofuranosidase AlFFase3 demonstrated an optimal reaction pH of 6.5, which is higher than that of other *Aspergillus* β-D-fructofuranosidases, such as those from *A. thermomutatus* [9], *A. terreus* [22], and *A. niveus* [25], but similar to that of the enzyme from *A. tamarii*. It was also very similar to the β-D-fructofuranosidases from bacteria, such as *Bifidobacterium* spp. and *Bacillus* spp. [14,19,26,27,28]. Furthermore, AlFFase3 exhibited excellent pH stability within the pH range of 5.0–8.0, which is distinct from other *Aspergillus* β-D-fructofuranosidases, such as those from *A. sojae* (pH 3.0–6.0) [15] or *A. niveus* (pH 2.0–8.0) [25], but comparable to the enzymes from *Bifidobacterium* spp. (pH 5.5–7.5) [29,30].

The optimal temperature for AlFFase3, a β-D-fructofuranosidase, was determined to be 40 °C, indicating that this enzyme can exhibit its maximum catalytic properties under low-energy-consumption conditions. In contrast, several other *Aspergillus* β-D-fructofuranosidases, such as those from *A. thermomutatus* [9], *A. sojae* [15], and *A. terreus* [22], require higher temperatures, with maximal activity at 60 °C. Moreover, AlFFase3 exhibited stability at temperatures up to 45 °C, which is similar to several other *Aspergillus* β-D-fructofuranosidases [9,25], but more stable than the β-D-fructofuranosidases from *A. sojae* [15] and *Gongronella* sp. w5 [31].

**Figure 4:**
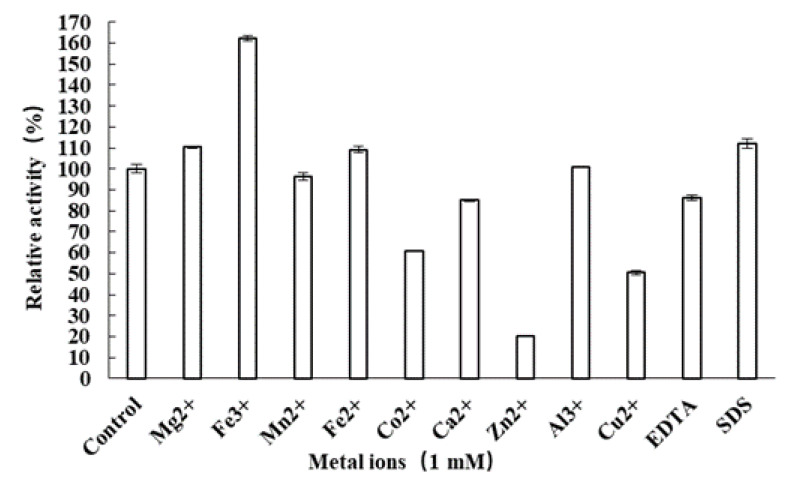
Effect of various metal ions on the β-D-fructofuranosidase of AlFFase3.

In practical biotechnological applications, the presence of metal ions can significantly affect the activity of commercially applied enzymes. Our results demonstrate that AlFFase3 activity is influenced by most tested cations, as well as SDS and EDTA (Figure 4). Interestingly, 1 mM Fe^3+^ and 1 mM Mg^2+^ effectively activated AlFFase3, whereas 10 times this amount of each was required to activate the β-D-fructofuranosidase from *A. sojae* [15]. Additionally, Fe^2+^ was found to activate AlFFase3, as well as the β-D-fructofuranosidases from *A. sojae* [15] and *A. niveus* [25], but not other enzymes, such as FTase [2]. However, Zn^2+^ strongly inhibited the activity of AlFFase3, which is a common inhibitor of many β-D-fructofuranosidases [29]. Cu^2+^ also significantly inhibited the activity of AlFFase3, as well as other microbial β-D-fructofuranosidases, such as InvDz13 from *Microbacterium trichothecenolyticum* [19] and GspInv from *Gongronella* sp. W5 [31]. This could be due to the ability of Cu^2+^ to catalyze the auto-oxidation of cysteine, leading to the inactivation of various glycoside hydrolases through the formation of intramolecular disulfide bridges or sulfenic acid [32]. Interestingly, the chelating agent EDTA did not affect the β-D-fructofuranosidase from *A. thermomutatus* [9], but it inhibited the activity of AlFFase3. Taken together, these findings suggest that AlFFase3 is a metal-dependent enzyme. Notably, AlFFase3 demonstrated increased activity in the presence of SDS, whereas the β-D-fructofuranosidase from *A. sojae* was inhibited by SDS [15]. This unique characteristic may make AlFFase3 valuable in various industrial applications.

### 3.4. Resistance of AlFFase3 to Proteases

**Figure 5:**
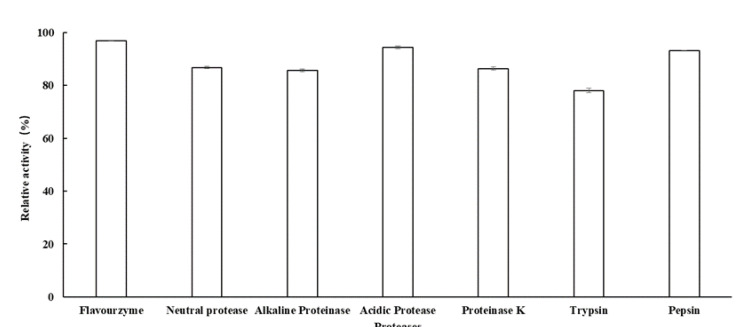
The ability of β-D-fructofuranosidase AlFFase3 to resist various proteases.

AlFFase3 demonstrated high resistance to most of the tested proteases, including Flavourzyme (96.8%), acidic protease (94.4%), pepsin (93.2%), neutral protease (86.7%), Proteinase K (86.4%), alkaline proteinase (85.7%), and trypsin (78.1%) (Figure 5). In the food-processing industries, glycoside hydrolases are often used in combination with proteases to produce more digestible end products from protein-rich materials. The soluble protein AlFFase3 exhibited good resistance to commonly used proteases, possibly due to the presence of various sites for proteolytic hydrolysis that are concealed inside the structure of AlFFase3. There is limited information available in the literature regarding the resistance of other β-D-fructofuranosidases to proteases; however, similar observations have been reported for xylanase and glucosidase [33,34,35].

### 3.5. New Function of Producing Various FOSs of AlFFase3

**Figure 6:**
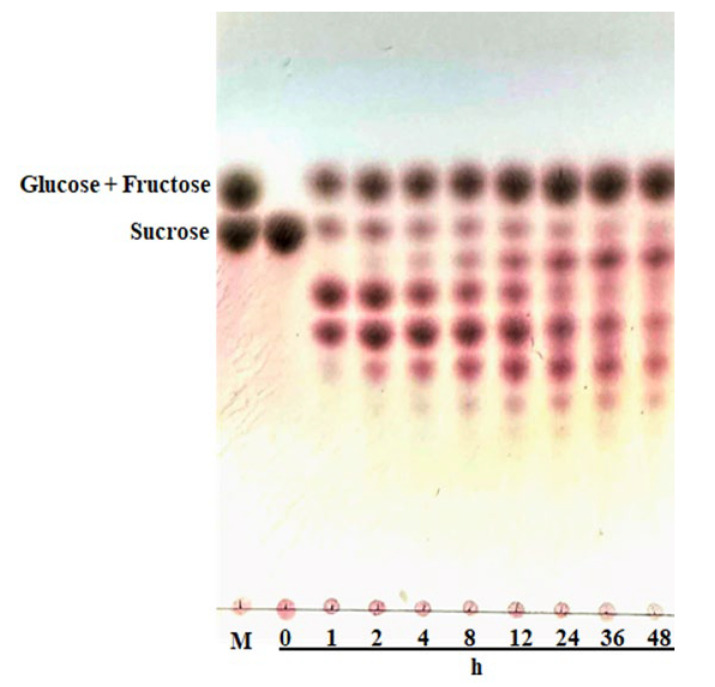
Potential application of AlFFase3 in FOS preparation by AlFFase3. The reaction mixture, containing 200 mg/mL of sucrose in 50 mM sodium phosphate buffer (pH 6.5) and 10 U/mL of AlFFase3, was incubated at 40 °C. Lane M—a mixture of fructose, glucose and sucrose; the other lanes—end products from hydrolysis of sucrose at different times.

A new function of AlFFase3 has been discovered, namely significant transfructosylation activity leading to the production of various fructooligosaccharides (FOSs) that are widely used in the food industry (as shown in Figure 6). The FOS yield obtained in this study exceeded 67%, and FOSs with varying degrees of polymerization (DP) were produced, including trisaccharides, tetrasaccharides, pentasaccharides, and hexasaccharides. During the initial stage of the reaction, tetrasaccharides and pentasaccharides were predominantly produced, whereas trisaccharides were the main end products towards the end of the reaction. This discovery opens up new possibilities for the application of AlFFase3 in the food industry.

In the industry, the production of fructooligosaccharides (FOSs) can be achieved through the hydrolysis of inulin or by the transfructosylation of sucrose. The use of sucrose is advantageous due to its lower cost compared to inulin [7]. Although transfructosylation is a common phenomenon in glycoside hydrolases, the study of the transfructosylation activity of β-D-fructofuranosidases is still limited. β-D-Fructofuranosidases from various taxa, including *Aspergillus*, *Pichia pastoris*, and *Aureobasidium melanogenum*, have been found to exhibit transfructosylation activity [2]. Fortunately, AlFFase3 from *A. luchuensis* has shown high transfructosylation activity, which is also the first report for this species, indicating its potential for application in FOS preparation. Additionally, the yield of FOSs produced by AlFFase3 exceeded 67%, which is similar to the β-D-fructofuranosidase from *A. oryzae* N74 (70%), but higher than all other reported β-D-fructofuranosidases [36]. Generally, the β-D-fructofuranosidases mentioned above exhibit both hydrolytic and transfructosylation activities, with the extent of the two activities depending not only on the amount of the enzyme, but also on the substrate concentration. The transfructosylation action typically occurs at high concentrations of sucrose, as the high content of sucrose in the system decreases the water activity and orients the reaction towards transfructosylation [7]. The transfructosylation reaction consists of the transfer of a sugar residue onto the substrate itself (auto condensation) or onto another hydroxylated molecule present in the enzyme’s active site [4]. During the reaction of AlFFase3, the sucrose concentration continuously decreased, indicating that the hydrolysis of sucrose by AlFFase3 is not associated with the ping-pong mechanism reported for fructosyltransferases [7]. Figure 6 shows that AlFFase3 produced FOSs from DP3 to DP6, suggesting the potential to achieve high yields of FOSs in industrial production. In the initial stage of the reaction, monosaccharides and FOSs with various DPs increased along with the rapidly decreasing sucrose concentration, indicating that the transfructosylation activity of AlFFase3 is dominant at first [7]. As the reaction time increased, the hydrolytic activity became more pronounced, and the initially produced FOSs with high DP degraded into products with low DP. For industrial purposes, FOSs with low DP are desirable because of their better therapeutic properties when compared with molecules with high DP [9].

### 3.6. Effect on Gel Properties of AlFFase3 during the Processing of Yoghurt

**Figure 7:**
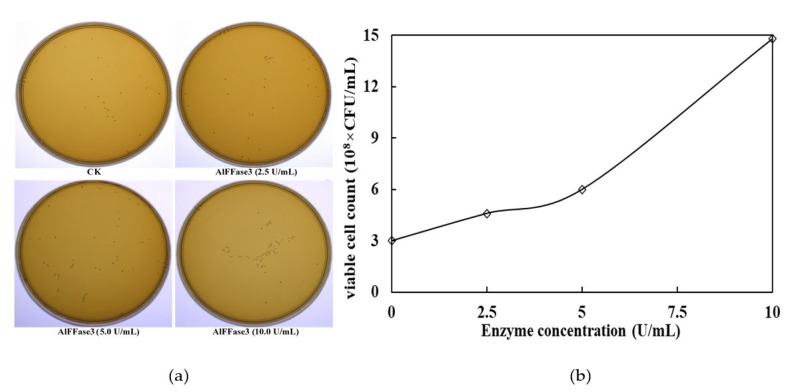
Effect of AlFFase3 on the proliferation of probiotics during yogurt fermentation process ((**a**), colony growth images; (**b**), viable cell count results).

**Figure 8:**
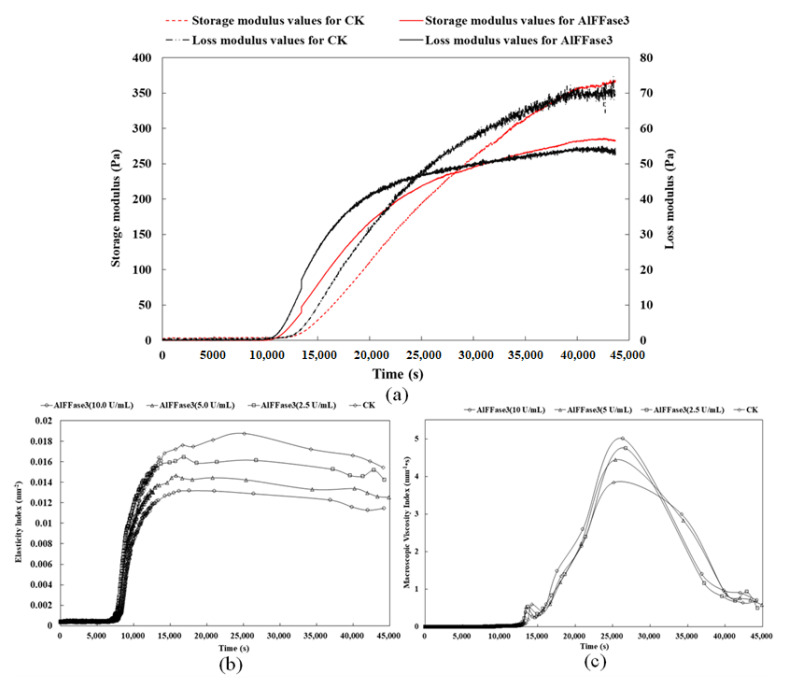
Effect of AlFFase3 on the gelation period ((**a**), Storage and loss modulus and yogurt gel characteristics; (**b**), Elasticity Index; (**c**), Macroscosity Index) during yogurt fermentation.

This study demonstrated that AlFFase3 could promote the proliferation of probiotics during yogurt fermentation. An addition of 10 U/mL of the enzyme resulted in an increase in the number of viable bacteria from 3 × 10^8^ CFU/mL to 1.5 × 10^9^ CFU/mL (Figure 7). Furthermore, AlFFase3 could effectively improve the formation pattern and properties of yogurt gel during fermentation. On the one hand, adding 10 U/mL of β-D-fructofuranosidase can simultaneously shorten the time for yogurt to start forming a gel, and increase the stability of the gel (Figure 8a). Incorporating A1FFase3 during yogurt fermentation can effectively decrease the onset time of yogurt gelation from around 3.3 h to 2.75 h, and shorten the time for the formation of stable yogurt gel from 11.1 h to 7.2 h. On the other hand, AlFFase3 can also regulate some properties of yogurt gel. For example, the elasticity of the gel decreases with increasing enzyme concentration (Figure 8b), while the viscosity of the gel increases with increasing enzyme concentration (Figure 8c).

The addition of AlFFase3 to yogurt has resulted in a significant increase in the number of probiotics, which can be attributed to its ability to hydrolyze sucrose into glucose and fructose, providing a more suitable energy source for the growth of probiotics. Moreover, AlFFase3′s strong transglycosylation ability as a β-D-fructofuranosidase allows it to fully utilize sucrose substrate and synthesize high yields of FOSs during yogurt fermentation. FOSs, which are typical prebiotics, have been shown to promote the rapid growth of probiotics and other beneficial microorganisms, and their ability to continuously accumulate more FOSs during fermentation further enhances the proliferation of probiotics, accelerating the acidification rate of yogurt fermentation and shortening the period needed for yogurt gel formation and stabilization. Obviously, the addition of AlFFase3 has altered the sugar composition in yogurt fermentation, which may be one of the reasons it improves the elasticity of yogurt gel. For example, studies have suggested that certain FOSs can form clusters, which can regulate the protein network structure of casein micelles during yogurt fermentation, affecting the elasticity and viscosity of yogurt gel [37]. Furthermore, FOSs have the advantages of being low in calories and thermally stable in food processing. Evidently, the application of A1FFase3 can not only boost the nutritional value of yogurt by augmenting the levels of prebiotics and probiotics in the yogurt, but also shorten the period needed for yogurt gel formation and effectively improve the characteristics of yogurt gel, highlighting the excellent food-processing advantages of this enzyme.

## 4. Conclusions

In conclusion, this study describes the expression and characterization of a chemically synthesized β-D-fructofuranosidase gene, AlFFase3, from *A. luchuensis* in *E. coli*. The enzyme exhibits high substrate affinity, stability, and protease resistance, and possesses both hydrolytic and transfructosylation activities, making it a promising candidate for FOS production. Notably, the recombinant β-D-fructofuranosidase demonstrates potential applications in the processing of yogurt, as it improves the nutritional value and gel characteristics of the yogurt, while also shortening the gel formation period. In the future, the abundant properties of AlFFase3 will serve as a theoretical foundation for its wider application in the food industry. For instance, this enzyme could be employed to transform sucrose in alternative foods, generating FOSs that can be utilized in probiotic-fermented foods and the like.

## Figures and Tables

**Table 1:** Summary of the recombinant β-D-fructofuranosidase (AlFFase3) expressed in *E. coli*.

Purification Step	Total Activity	Protein	Specific Activity	Purification	Recovery
	(U) ^a^	(mg) ^b^	(U/mg)	Factor (-Fold)	(%)
crude supernatant	20381.1	147.4	138.2	1.0	100.0%
Ni-IDA	8032.2	10.4	771.2	5.6	39.4%

^a^ Activity was measured in 50 mM phosphate buffer (pH 6.5) at 40 °C using 20% sucrose. ^b^ The protein was measured by the Lowry method, using BSA as the standard.

## Data Availability

Not applicable.
